# Unlocking the Potential of Insect-Based Proteins: Sustainable Solutions for Global Food Security and Nutrition

**DOI:** 10.3390/foods13121846

**Published:** 2024-06-12

**Authors:** Hugo M. Lisboa, Amanda Nascimento, Amélia Arruda, Ana Sarinho, Janaina Lima, Leonardo Batista, Maria Fátima Dantas, Rogério Andrade

**Affiliations:** Unidade Academica Engenharia de Alimentos, Universidade Federal Campina Grande, Av. Aprigio Veloso, 882, Campina Grande 58429-900, PB, Brazil

**Keywords:** edible insects, sustainable protein, food security

## Abstract

The present review highlights the potential of insect-based proteins to address the growing need for sustainable and secure food systems. The key findings suggest that edible insects offer a viable and environmentally friendly alternative to traditional livestock, requiring significantly less land, water, and feed while emitting lower levels of greenhouse gases. Insect farming can also reduce waste and recycle nutrients, supporting circular economy models. Nutritionally, insects provide high-quality protein, essential amino acids, and beneficial fats, making them valuable to human diets. Despite these benefits, this review emphasizes the need for comprehensive regulatory frameworks to ensure food safety, manage potential allergenicity, and mitigate contamination risks from pathogens and environmental toxins. Additionally, developing innovative processing technologies can enhance the palatability and marketability of insect-based products, promoting consumer acceptance. This review concludes that with appropriate regulatory support and technological advancements, insect-based proteins have the potential to significantly contribute to global food security and sustainability efforts.

## 1. Introduction

The global food system faces mounting challenges as it strives to meet the nutritional demands of an ever-growing population amidst the constraints of environmental sustainability [1]. Traditional livestock production, a primary source of protein, is associated with significant environmental impacts, including high greenhouse gas emissions, extensive land and water use, and substantial contributions to deforestation and biodiversity loss [2]. These challenges necessitate exploring alternative, sustainable protein sources to alleviate the strain on natural resources while ensuring food security.

Edible insects have emerged as a promising solution to these challenges, offering a sustainable and nutritious alternative to conventional animal proteins. Fifty years ago, Meyer-Rochow suggested that insects could help ease the potential problem of world food shortage [3]. Insects are highly efficient at converting feed into protein, require minimal land and water, and can be reared on organic waste streams, thereby contributing to waste reduction and nutrient recycling within circular economy models [4]. Nutritionally, insects are rich in high-quality protein, essential amino acids, and beneficial fats, making them a valuable addition to human diets [5].

Despite the recognized benefits, several barriers hinder the widespread adoption of insect-based proteins. There is a notable gap in comprehensive regulatory frameworks to ensure the safety and quality of insect-derived food products [6]. Potential risks must be systematically addressed, such as contamination with pathogens, heavy metals, and allergens [7]. Moreover, consumer acceptance remains a significant challenge, particularly in Western societies, where entomophagy is not traditionally practiced [8]. Innovative processing technologies and effective marketing strategies are essential to enhancing the palatability and appeal of insect-based products [9,10]. Furthermore, ethical and ecological considerations are increasingly important. Many countries have implemented legal protections for insect species, including those traditionally collected for food and therapeutic purposes [11,12]. For example, in Japan, collecting the giant waterbug is illegal, reflecting broader trends in protecting insects from cruelty and inhumane treatment. Animal protection organizations are likely to advocate for such measures, bringing ethical considerations to the forefront. Additionally, the ecological roles of insects must be considered [13]. For instance, dragonfly nymphs collected in northeastern India play crucial roles in controlling pest populations, and their removal can significantly impact local ecosystems [14].

The study on insect-based proteins presents a compelling case for their integration into the global food system, offering sustainable and nutritious alternatives to conventional protein sources. The potential of insect-based proteins generates significant multiplier effects at both regional and global levels. By incorporating insect proteins into the food supply, regions can reduce dependence on traditional livestock, decrease greenhouse gas emissions, and conserve water and land resources, thereby supporting the circular economy through the utilization of organic waste streams as insect feed. This transition can stimulate local economies by creating job opportunities in farming, processing, and distribution while enhancing food security with reliable, locally sourced protein alternatives. Domestication and semi-domestication of insect species ensure sustainable and controlled farming practices, providing consistent supply and quality. Globally, integrating insect-based proteins can address the growing protein demand, particularly in regions facing food shortages and malnutrition, fostering a shift towards resilient and diverse food systems. These multiplier effects highlight the transformative potential of insect-based proteins in promoting sustainable development and global food security.

This review aims to bridge these research gaps by thoroughly examining the current state of insect-based protein production, processing technologies, and safety considerations. The objectives of this review are threefold:

- Assess the environmental and nutritional advantages of incorporating insect-based proteins into the global food system.
- Evaluate the existing regulatory frameworks and identify areas requiring further development to ensure the safe consumption of insect-derived foods.
- Explore consumer perceptions and acceptance of edible insects, highlighting strategies to increase their marketability and integration into mainstream diets.

By addressing these objectives, this review seeks to provide a comprehensive overview of the potential of insect-based proteins to contribute to a more sustainable and secure food system, emphasizing the need for regulatory advancements and consumer education to facilitate their broader adoption.

## 2. Materials and Methods

### 2.1. Literature Review

A comprehensive literature review was conducted to assess the potential of insect-based protein for a sustainable and secure food system. The review included peer-reviewed articles, books, reports, and other scientific publications from reputable sources. The databases used for this review included Google Scholar, PubMed, and Scopus. The search terms included “insect-based protein”, “edible insects”, “sustainable food systems”, “entomophagy”, “insect farming”, and “food security”.

### 2.2. Data Collection

The nutritional content of edible insects was extracted from various studies and compiled into tables. This included information on protein content, fat content, essential fatty acids, vitamin B12, iron, and zinc levels. The sources for these data are cited in the References section.

### 2.3. Analysis

The collected data were analyzed to perform the following:

- Evaluate the environmental impact of insect farming by assessing greenhouse gas emissions, land and water usage, and the ability of insects to convert organic waste into protein.
- Examine the economic viability by evaluating the costs of insect farming, potential market growth, and economic benefits for smallholder farmers.
- Investigate the nutritional benefits by determining the macronutrient and micronutrient profiles of various insect species compared with traditional livestock to highlight their nutritional advantages.
- Potential risks such as contamination with pathogens, heavy metals, and allergens were reviewed. Strategies to mitigate these risks through controlled farming practices and thorough processing methods were also examined.

### 2.4. Case Studies

Global initiatives and brands pioneering the use of insect-based proteins were reviewed. These case studies provided practical examples of the commercial viability and innovative approaches to insect farming and processing.

## 3. Sustainability of Insect-Based Protein

### 3.1. Environmental Impact

Traditional livestock, particularly ruminants, are major producers of methane and have become a major concern regarding climate changes due to greenhouse gas emissions [15]. Ruminants such as cows and sheep, known for their enteric fermentation process, are significant contributors to global methane emissions [16]. This digestive process is responsible for a considerable proportion of the greenhouse gases attributed to livestock, which in turn account for about 14.5% of global emissions. Research suggests that implementing strategies like dietary modifications, genetic selection, and improved management practices can effectively reduce methane emissions from these animals [17,18]. These efforts are essential to reducing the environmental impact of livestock and addressing climate change effectively.

Insect farming presents a significant opportunity for reducing agriculture’s impact on climate change, primarily due to insects’ lower greenhouse gas emissions than traditional livestock. Studies indicate that insects such as crickets emit drastically less methane than cattle—up to 80 times less per kilogram of body weight [19]. This stark contrast underscores the potential environmental benefits of shifting from cattle to insects for protein production. Not only do insects offer a more sustainable option in terms of emissions, but they also require less land and water, further enhancing their appeal as an alternative protein source. Ref. [20] explored the potential of edible insects as a sustainable alternative to traditional livestock, focusing on greenhouse gas (GHG) and ammonia emissions. Through experiments on five insect species, the study found that insects have lower GHG and ammonia emissions compared with conventional livestock such as cattle and pigs. Additionally, insects showed higher growth rates and more efficient feed conversion. The findings suggest that insects could serve as a more environmentally friendly source of animal protein, offering significant reductions in GHG emissions and other environmental impacts associated with traditional livestock farming. Similarly, Boakye-Yiadom and co-workers [21] reviewed greenhouse gas emissions from black soldier fly larvae (BSFL) during bioconversion, highlighting that BSFL processes emit relatively low levels of carbon dioxide (CO_2_), methane (CH_4_), and nitrous oxide (N_2_O), primarily due to the semi-aerobic conditions of their rearing environment. Ammonia (NH_3_) emissions are noted but decrease over time as the organic matter is degraded. Overall, BSFL bioconversion presents a sustainable alternative to traditional livestock and waste management practices, with lower associated greenhouse gas emissions.

Livestock farming significantly impacts deforestation, especially in regions where large tracts of forest land are cleared for grazing and to cultivate feed crops [22]. This practice not only leads to a loss of biodiversity by destroying habitats for countless species but also increases carbon emissions. Deforestation is the second largest anthropogenic source of carbon dioxide emissions, with carbon-focused policies potentially offering lower benefits for biodiversity than policies with a more biodiversity-focused approach [23]. There is a strong association between carbon stocks and species richness, suggesting that carbon-based conservation could protect areas of high value for biodiversity, but some regions may not benefit from it and could face increased pressure if REDD (Reducing Emissions from Deforestation and Forest Degradation) is implemented without considering biodiversity distribution [24]. Additionally, the loss of forests diminishes the planet’s capacity to absorb CO_2_, further hindering efforts to mitigate global warming. This cycle of deforestation and its environmental impacts underscore the critical need for sustainable practices in agriculture and animal husbandry to protect natural ecosystems and combat climate change [25].

Insect farming presents a highly sustainable alternative to traditional livestock due to its minimal land requirements [26]. Insects can be reared in vertically stacked containers, drastically reducing the land footprint compared with conventional animal farming [27]. For example, producing 1 kg of protein from insects like crickets requires significantly less land than producing the same amount of beef protein [28]. Insect farming is emerging as a sustainable alternative to conventional livestock production, addressing the increasing demand for food amidst limited agricultural land and the need to mitigate the impacts of climate change [29]. Insects are an efficient option, as they require significantly less land and water, emit lower levels of greenhouse gases, and can convert low-value organic by-products into high-quality food or feed.

Research in this field has revealed that insect farming possesses a smaller ecological footprint compared with traditional livestock farming, requiring fewer resources and producing fewer emissions. Moreover, conventional farming practices have been linked to biodiversity loss and the decline in ecosystem services. In contrast, urban and peri-urban agricultural practices, including insect farming, can help mitigate these issues [30]. Specifically, urban agriculture, which may incorporate insect farming, supports biodiversity and provides ecosystem services such as biological control, potentially reducing the reliance on chemical pesticides [31]. Furthermore, insect farming enhances sustainable urban food systems by recycling nutrients from organic waste back into the food chain [32]. Consequently, it not only minimizes the environmental impact of agriculture but also bolsters food security and supports vital ecosystem services, making it a particularly viable and environmentally friendly option for food production in urban areas where space is constrained and the demand for sustainable practices is heightened.

Water scarcity is a pressing environmental issue, especially in the context of traditional livestock farming, which requires substantial water resources. In contrast, insects provide a more water-efficient alternative for food and feed production. In-depth research has indicated that insect farming demands significantly less land and water than traditional livestock production and generates lower greenhouse gas emissions [29]. Housefly larvae have the potential to convert waste into livestock feed, reducing the environmental impact of the livestock sector, although this process may increase energy use and greenhouse gas emissions depending on the waste treatment method [33]. Livestock farming, particularly intensive agricultural activities, has a negative impact on soil and water quality, with pig farms notably affecting surface water quality more than other livestock farms [34]. Water scarcity is a regional issue with global implications, and minimizing the water footprint of livestock can be achieved by reducing the irrigation of feed crops [35]. Moreover, cattle farming can degrade water quality and negatively affect aquatic ecosystems, including amphibian populations [36]. Minilivestock ranching, such as insect farming, can significantly reduce the carbon footprint and the negative environmental impacts of agriculture, offering a much lower water requirement [37].

Insect farming is promoted as a sustainable practice due to its limited need for arable land and water, supporting circular economic processes [38]. Additionally, societal expectations in Europe show a preference for addressing the environmental impacts of livestock farming, with public willingness to pay for mitigation efforts [39].

Insects convert feed into edible body mass much more efficiently than livestock. This is because insects are cold-blooded and do not need to expend energy to maintain body heat. For instance, crickets require only 1.7 kg of feed to produce 1 kg of meat, whereas cattle may need up to 8 kg of feed for the same amount of meat. Insects, known for their high feed conversion ratios, can transform low-value organic wastes into high-quality feed, potentially replacing fishmeal and soybean meal in livestock diets [40,41]. While genetic improvement in cattle feed efficiency exists, particularly by selecting for traits like residual feed intake (RFI), the benefits may be limited compared with the potential offered by insect-based feed [42]. Furthermore, nearly the entire insect is edible, unlike livestock, where significant portions of the body (like bones and fur) are not consumed.

Insects, especially Orthoptera species, exhibit much lower feed conversion ratios compared with traditional livestock, suggesting that a shift towards insect protein could lessen greenhouse gas emissions and reduce the use of arable land for feed production [43]. Although selection for low RFI in cattle can decrease methane emissions per unit of product, the overall impact on greenhouse gas emissions may be less significant than adopting insect-based feed [44]. Furthermore, the mass rearing of edible insects like field crickets has proven feasible under commercial conditions, with efficient nutrient conversion rates, supporting the viability of insects as a sustainable feed source [45]. Insect farming also contributes to waste reduction, since many insects can be fed organic waste streams such as food scraps, reducing waste and the need for disposal [46].

An important ecological consideration is the impact of harvesting predatory insect species. Predatory insects, such as dragonflies and certain beetles, play a crucial role in maintaining the balance of ecosystems by controlling pest populations [47]. The removal of large numbers of these predatory species can disrupt local ecosystems, leading to an increase in pest populations and subsequent ecological imbalances [48]. For example, dragonfly nymphs, often harvested in northeastern India, are significant predators of mosquitoes and other insects. Overharvesting these predators can lead to increased pest populations, potentially affecting human health and agricultural productivity [49].

### 3.2. Economic Viability

The economic viability of insect farming is gaining attention as an innovative solution to the escalating demands of sustainable food production. This form of agriculture offers several economic advantages due to its efficiency and scalability, and it integrates well with the principles of the circular economy. Additionally, insect farming is emerging as a sustainable and economically viable solution to meet the increasing demands for food and feed, aligning with the principles of the circular economy and addressing global food security challenges.

Insect farming requires less arable land and water, has a low ecological cost, and provides high-quality protein, making it a sustainable food source with a low environmental impact [38,50]. However, it is important to consider the economic challenges faced by insect-rearing facilities, particularly in temperate countries. These facilities often rely heavily on electricity for heating and lighting the breeding rooms or containers, significantly increasing operational costs [51]. Unlike tropical settings, where the natural climate is more conducive to insect rearing, temperate regions must invest in climate control to maintain optimal insect growth and reproduction conditions. The growth of insect farming in East Africa demonstrates its potential as a profitable enterprise, offering “climate-smart” protein and aligning with circular economy principles [52,53]. Inclusive business models involving insect farming can improve livelihoods for smallholder farmers, with low initial capital investments and contributions to food security and circular economy principles [54]. Insect rearing offers significant benefits to the agri-food industry and is sensitive to circularity and sustainability innovation, with potential growth in aquaculture [51].

The use of insects as livestock feed can improve sustainability by transforming low-value organic wastes into high-quality feed, reducing the environmental footprint of livestock production [40]. Regulatory frameworks, consumer education, and marketing strategies are crucial for the growth of the edible insect industry, especially in Western societies, where acceptance is low [55].

The scalability of insect farming is one of its most compelling economic benefits. Insects can be farmed in a variety of environments, from rural to urban settings, and they require significantly less space due to their ability to be farmed vertically [56]. This method of farming can be modular, allowing for small-scale operations to expand as demand increases. The adaptability of insect farming operations also means that they can be integrated into existing agricultural or food production systems, potentially reducing waste and increasing efficiency [57].

Insect farming fits well within the circular economy model by contributing to the sustainability of food systems. Insects can be fed bio-waste, such as food scraps and agricultural by-products, reducing waste and minimizing the dependency on traditional, resource-intensive feedstocks like soy meal and fishmeal. This waste-to-protein conversion addresses waste management issues while producing valuable food resources. Furthermore, insect frass (waste) can be used as organic fertilizer, contributing to soil health and reducing the need for chemical fertilizers.

### 3.3. Market 

The market for insect-based products is growing, driven by the rising consumer interest in sustainable and alternative protein sources. Products ranging from whole insects for human consumption to insect-based animal feed and pet food are expanding. The versatility of insects as a source of protein, fat, and other nutrients means they can be incorporated into a wide variety of products, potentially opening up numerous market opportunities. Insect protein is emerging as a sustainable alternative to traditional animal-based proteins, with the potential to reduce the carbon footprint associated with European food consumption, particularly when insects are directly consumed or used in animal feed [58]. Consumer acceptance is critical for the commercialization of insect-based foods, with design interventions and information dissemination playing key roles in increasing willingness to incorporate insects into diets [59,60,61]. 

However, it is important to note that while there may be increasing interest in insect-based products in some countries, the market dynamics are not universally positive. In countries that traditionally consumed insects, such as Thailand, Laos, Japan, and Mexico, insect consumption is declining [62]. This decline can be attributed to various factors, including modernization, changes in dietary preferences, and urbanization. In some parts of India, laws have been passed that curtail the sale of insects at local markets, further impacting traditional insect consumption. Consequently, while markets for insect-based products may experience growth in certain regions, they are shrinking or facing significant challenges in others [62,63].

Information on the environmental and nutritional benefits of insect-based feed can significantly increase consumer acceptance and willingness to purchase and consume products from animals fed insects [60]. Non-hypothetical market studies suggest that consumers’ willingness to pay for insect-based products can be influenced by the type of product (carrier) and the provision of positive information about edible insects [61]. The use of insect meal in aquaculture is viewed favorably by consumers, who are willing to pay a premium for fish produced with sustainable insect-based feed, despite lower taste expectations [64].

Research on animals fed insect-based diets indicates that insect-derived products can be a viable alternative to conventional feed sources, with considerations for digestibility, performance, and product quality [65]. In low-income countries, insect meal presents an opportunity for sustainable protein production, reducing dependency on imported feed and supporting local economies, in alignment with the United Nations Sustainable Development Goals [66].

In conclusion, insect-based products are gaining traction as a sustainable protein source with environmental and economic benefits. Consumer acceptance is pivotal and can be enhanced through strategic information and product design. The willingness to pay for such products is influenced by the product type and consumer education. Insect-based feed for livestock and aquaculture is promising, with research supporting its viability and consumer readiness to embrace these innovations.

### 3.4. Social Impacts

The adoption of insect farming holds the potential to exert significant social impacts, particularly in terms of cultural acceptance, economic opportunities in rural areas, and enhancements in food security. These impacts are interlinked with broader social, economic, and cultural frameworks that vary globally.

Smallholder farmers can benefit from low initial capital investments in insect farming, which can improve livelihoods and contribute to food security and a circular economy, aligning with the United Nations’ Sustainable Development Goals [54]. In Europe, the commercialization of edible insects is connected to cultural and regional values, and the industry’s growth could challenge current food systems, with research focusing on consumer acceptance and environmental impacts [67]. Biological control using insects can contribute to food security, poverty alleviation, and environmental preservation, highlighting the multifaceted role of insects in sustainable development [68]. The circular business model in insect farming emphasizes the need for economic promotion and research to enhance cost and ecological effectiveness, suggesting a focus on the entire supply chain [38].

Urban pest management strategies highlight the economic and social impact of social insects, with advances in control methods potentially reducing pesticide use and promoting environmental awareness [69]. Tsetse fly control in sub-Saharan Africa, involving the sterile insect technique, illustrates how insect management can catalyze rural development and alleviate hunger and poverty by enabling productive agriculture [70].

Entomophagy, the practice of eating insects, has been a part of human diets for millennia, particularly in parts of Africa, Asia, Latin America, and Oceania, where insects are often considered delicacies or staple foods due to their nutritional benefits, abundance, and cultural significance [71]. However, in Western societies, there is a cultural aversion to entomophagy, often seen as a disgusting practice, which presents a barrier to its acceptance as a sustainable and nutritious food source [72,73].

Entomophagy is recognized for its potential to address food demand and undernourishment, especially in underdeveloped countries, and is being reconsidered as a sustainable food source [72]. In Western societies, overcoming the disgust reaction and neophobia is crucial for the acceptance of entomophagy, which may involve disguising insects in food and emphasizing food safety [73]. Educational campaigns and improved knowledge about the preparation and nutritional benefits of insect-based foods could encourage Western seniors to adopt entomophagy.

Cultural and social stigmas against insect consumption are being challenged by promoting entomophagy as a sustainable and nutritious food source in Western societies [71]. Factors influencing the acceptance of entomophagy in Western cultures include taste, health perceptions, price, and knowledge about the environmental and nutritional advantages of insects [74,75]. Tradition and culture play a significant role in the perception and practice of entomophagy, with variations in knowledge and practices based on ethnicity, culture, age, and education [76,77]. Psychological factors such as attitudes, perceived behavioral control, and social norms, along with objective knowledge and culture, significantly influence entomophagy in regions where it is accepted, like western Kenya [78].

### 3.5. Population Dynamics and Food Security

While the global population continues to grow, many regions, particularly in Europe and Asia, are experiencing population declines [79]. For instance, in Europe, two-thirds of the EU region is projected to experience population decline by 2021 [80]. Similarly, East Asia is facing a looming demographic crisis with significant population reductions expected in countries like China and Japan [81]. These demographic changes pose challenges for food security and economic stability, as declining populations often lead to reduced agricultural labor forces and increased strain on food production systems [82].

Sustainable and efficient food sources like insect-based proteins become even more critical in this context. Insects require significantly less land, water, and feed than traditional livestock, making them an ideal protein source for regions facing population decline and resource scarcity [83]. Additionally, insect farming can be easily scaled up or down, providing flexibility to adapt to changing demographic trends and food demand.

Regions experiencing population decline can enhance their food security by incorporating insect-based proteins into their food systems [84]. Insects offer a reliable and resource-efficient protein source that can help stabilize food supplies, reduce dependence on imported proteins, and support local economies. Furthermore, insect farming aligns with the principles of a circular economy by utilizing organic waste streams, thus promoting sustainability and resilience in food production.

As the global population dynamics shift, adopting innovative and sustainable food sources like insect-based proteins will be crucial to ensuring food security and economic stability in the affected regions.

## 4. Nutrition 

Edible insects present a promising alternative source of macronutrients and micronutrients. They have the potential to contribute to human nutrition and address issues of food security and undernutrition, especially in food-insecure regions. The sustainability of insects as a food source, along with their nutritional benefits, makes them an attractive option for future food systems [85]. 

### 4.1. Macronutrients 

The nutritional composition of edible insects varies widely across species. There is a vast amount of detailed chemical analyses of edible insects in the literature. Studies by Ghosh et al. have provided comprehensive data on the chemical composition and nutritional value of various species, including Vespa hornets, honeybee brood, termites, beetles, and others [86,87,88,89,90,91,92].

Furthermore, the chemical composition, nutrient quality, and acceptability of edible insects are significantly affected by species, developmental stage, gender, diet, and processing method, as highlighted by Meyer-Rochow et al. (2021) [93]. Understanding these factors is crucial to optimizing the nutritional benefits of edible insects and ensuring their suitability as a food source.

Table 1 summarizes the nutritional content of five selected edible insect species. The species were chosen based on their expected availability for consumption and their representation in the literature as nutritionally significant.

Insects have been recognized as a significant source of macronutrients essential for the growth and development of various species, including humans. The nutritional composition of insects varies widely, but they generally provide a substantial amount of protein, fats, and carbohydrates, as well as micronutrients such as vitamins and minerals [100,101,102]. In the context of hunter–gatherer diets, insects have been proposed to serve as an invertebrate equivalent of vertebrate-derived meats, primarily as a source of protein. However, the macronutrient composition of insects is more varied than that of wild vertebrate meats, potentially allowing insects to serve as equivalents of a range of other foods, including shellfish, nuts, pulses, vegetables, and even fruits [103]. It is important to clarify that while insects are rich in high-quality protein and other nutrients, they are not universally more nutritious than conventional meats. Payne et al. (2016) critically analyzed the nutritional profiles of edible insects compared with commonly consumed meats and found that insects are generally lower in the essential amino acids methionine and tryptophan [104]. Therefore, while insects can be a valuable addition to the diet, they should be considered part of a diverse and balanced nutritional strategy rather than a direct replacement for conventional meats.

In aquaculture, insect meals have been shown to be a viable replacement for fish and soybean meals, providing high levels of indispensable amino acids, lipids, and fatty acids. The nutritional value of insect meals can be influenced by the species, developmental stage, and type of feed [105]. 

Whole insects are often consumed roasted or fried, providing distinctive textures and flavors. Insect powders serve as protein supplements in shakes, baking, or cooking, offering a palatable alternative for those unaccustomed to eating whole insects. Additionally, insect-based oils and fats can be extracted for use in cooking or as dietary supplements.

#### 4.1.1. Proteins and Amino Acids

Protein content in insects can range from 20 to 76% of dry matter, depending on the type and developmental stage of the insect. This high protein content makes insects a potential alternative to traditional sources of animal protein [106]. Further studies have shown that insects can contribute to human health by promoting growth, influencing iron status, modulating gut microbiota with prebiotic effects, and providing amino acids comparable to soy protein [107].

The work of [101] provides an extensive compilation of the nutritional composition of 236 edible insects, revealing significant variability in their protein content, which ranges from 7% to 91% dry weight, with an average of around 60% for most species [108]. Orthopterans, such as *Melanoplus mexicanus*, have been noted to contain up to 77% protein dry weight. Other insects, like larvae of *Tenebrio molitor*, adults of *Gryllus sigillatus*, and the desert locust *Schistocerca gregaria*, have reported protein contents ranging from 52% to 76% dry weight [109]. Even marketed edible insect products in the EU, like whole *Locusta migratoria* and house cricket powders, exhibit significant protein percentages, ranging from 46.8% to 59.5% dry weight [110].

Payne and co-workers [111] highlighted the extensive variability in protein content, which can vary by over 50% even within the same insect species. This variability is influenced by several factors, including the insects’ diet, ecological conditions, developmental stages, and processing methods. The traditional nitrogen-to-protein conversion factor of 6.25, typically used for estimating protein content via Kjeldahl analysis, has been found to overestimate protein levels in insects due to their high non-protein nitrogen content, largely attributed to chitin. A revised conversion factor of 4.76 for larvae of *T. molitor*, *A. diaperinus*, and *H. illucens* has been suggested for more accurate protein quantification [112]. 

In terms of amino acids, edible insects are rich in essential amino acids, which are crucial for human nutrition. These include Isoleucine, Leucine, Lysine, Methionine, Phenylalanine, Threonine, Tryptophan, and Valine. Many insect species feature amino acid profiles that meet or exceed the requirements outlined by the FAO and WHO for infants [113,114]. For example, the amino acid indices in insects like *T. molitor*, *Zophobas morio*, and *Alphitobius diaperinus* are comparable to those found in recognized protein sources like soybean and bovine caseins [115].

Additionally, studies on the digestibility of insect proteins suggest high values, which is critical for their potential integration into human diets. Digestibility reports range from 54% for *T. molitor* to up to 98% for some species studied in Mexico [116].

However, despite these promising attributes, identifying and characterizing the proteins in edible insects remain challenging tasks due to incomplete proteomic data and the complexity of their protein structures. Advances in proteomic analysis, particularly through techniques like LC-MS/MS, are essential to better understanding and utilizing these proteins in food science and nutrition [117]. This continuous exploration is vital to fully unlocking the potential of edible insects as a protein-rich food source in sustainable diets globally.

#### 4.1.2. Fat 

The fat content in insects also shows considerable variability, ranging from 2 to 50% of dry matter. Notably, polyunsaturated fatty acids (PUFAs) can constitute up to 70% of the total fatty acids in some insect species, which is beneficial for human health [118]. It is important to note that not all saturated fatty acids are “bad”. For example, stearic acid, a type of saturated fatty acid commonly found in insects, has been shown to have a neutral or even beneficial effect on cholesterol levels. According to Bonanome and Grundy (1988), dietary stearic acid does not increase plasma cholesterol and lipoprotein levels, unlike other saturated fatty acids, and can help reduce LDL cholesterol levels [119]. This highlights the complexity of dietary fats and the need to consider the specific types of fatty acids present in insect-derived fats when evaluating their health impacts. 

Edible insects are recognized for their significant fat content, which varies greatly among different species and stages of development. According to the literature, the fat content in insects can range from 8% to 70% based on dry weight, with the fatty acid profiles of insects showing similarities across various meat sources, including different insect groups [120]. Particularly noteworthy are the larvae of Lepidopterans and Heteropterans, which exhibit higher fat contents than other edible insects, making them an excellent source of fatty acids or oils. In contrast, adult insects generally have a lower fat content, typically less than 20%.

The fat in insects primarily consists of triacylglycerol [121], and their fatty acid composition is predominantly made up of saturated fatty acids (SFAs) and monounsaturated fatty acids (MUFAs), which together constitute over 80% of the total fats. SFAs in insects, primarily composed of palmitic acid and stearic acid, are usually more prevalent in adults than MUFAs, which are considered healthier for human consumption. Oleic acid, a common MUFA in the human diet, is abundant in insects and is noted for its potential health benefits, including reducing blood pressure and aiding in the treatment of inflammatory, immune, and cardiovascular diseases [122]. 

However, mature insects are particularly valued for their rich content of polyunsaturated fatty acids (PUFAs), surpassing traditional sources like pork and beef. Linoleic acid, the predominant PUFA in insects, has anti-inflammatory properties and benefits for skin health, such as reducing acne and lightening the skin tone. Orthopterans are identified as the best source of linoleic acid among insect orders. Additionally, Lepidopterans are particularly high in α-linolenic acid, recognized for its nutraceutical potential for protecting the brain from stroke [123].

Both linolenic acid and α-linolenic acid are essential for humans, as they cannot be synthesized internally and must be obtained through the diet. They serve as precursors for the synthesis of prostaglandins, thromboxanes, and leukotrienes, which are crucial to maintaining normal visual functions. Insufficient intake of these fatty acids can lead to growth retardation, reproductive issues, skin disorders, and diseases affecting the kidneys, the liver, and the neurological and visual systems in humans. This highlights the importance of including adequate amounts of these essential fatty acids in the diet, for which edible insects can serve as an excellent source.

#### 4.1.3. Carbohydrates and Fibers 

Carbohydrates in insects are mainly represented by chitin, a polysaccharide that also serves as a source of dietary fiber. Chitin is found in the exoskeletons of insects and is structurally similar to cellulose, with an amine group shielded by the acetyl group [124]. The chitin content in insects can vary significantly, and a report suggested that it could be digestible by humans [125], so it may have nutritional and health benefits beyond improving gut health as a prebiotic [126]. 

Chitosan, derived from the deacetylation of chitin, has been studied for its prebiotic properties and its ability to promote the growth of beneficial probiotic microorganisms in the human gut. An important publication by Kipkoech et al. (2021) highlights the potential of cricket chitosan as a prebiotic. The study demonstrated that cricket chitosan could support the growth of probiotic microorganisms, such as *Lactobacillus* and *Bifidobacterium*, while inhibiting pathogenic bacteria like *Escherichia coli* and *Salmonella* [127]. This suggests that incorporating insect-derived chitosan into the diet could help improve gut health by enhancing the beneficial microbiota and controlling harmful bacteria.

### 4.2. Micronutrients 

#### Minerals and Vitamins

Edible insects are increasingly recognized as a significant source of nutrition and a potential solution to food security challenges. These insects are rich in a variety of micronutrients that are crucial for human health, including potassium, sodium, calcium, copper, iron, zinc, manganese, phosphorus, magnesium, and selenium. Their content in vitamins such as A, B1-12, C, D, E, and K varies among different species and is influenced by factors such as diet, seasonality, and environment [100,101].

For example, caterpillars are notably rich in vitamins B1, B2, and B6, while bee brood (pupae) is a good source of vitamins A and D [128]. The red palm weevil (*Rhynchophorus ferrugineus*) stands out for its high vitamin E content [120]. Additionally, insects like mealworms, crickets, grasshoppers, and cockroaches are validated sources of vitamin B12, which is essential for nerve function and blood formation. Crickets, for instance, contain approximately 2.88 µg of vitamin B12 per 100 g, and cockroaches have the highest level, 13.2 µg per 100 g [129]. Notably, the majority of vitamin B12-like substances in commercially available cricket products are actually pseudovitamin B12 and factor S, with authentic vitamin B12 constituting only a small percentage of the total corrinoids [130]. Given the variability in micronutrient content, a diverse diet or supplementation may be necessary to meet daily nutritional needs, especially for vegetarians or vegans who may be at risk of vitamin B12 deficiency [131]. 

Even though insects are rich in micronutrients such as vitamins and minerals, their contents can vary significantly among different insect species [132]. In a previous work [133], six insect species were analyzed, and *Bombyx mori* emerged with the highest calcium levels, almost on par with semi-skimmed cow’s milk. *Gryllus assimilis* was also proved to be a rich calcium source. In contrast, *Zophobas morio* showed the lowest calcium content. Notably, all the studied insect species, including *Apis mellifera*, *Locusta migratoria*, and *Tenebrio molitor*, displayed higher calcium levels than common meats such as chicken, beef, and pork. Additionally, phosphorus levels were measured, revealing *Bombyx mori* has the highest content, crucial for calcium–phosphate metabolism, and *Zophobas morio* presents the lowest content. 

Potassium and magnesium are other relevant minerals for human diet. It was previously reported that the pupae of *Polybia occidentalis* have a potassium content of 54 mg/100 g [134], while all stages of *Apis mellifera* offer at least 1500 mg of potassium per 100 g [135]. The magnesium content also shows dramatic variation; the adults of *Macrotermes nigeriensis* provide only 6.1 mg per 100 g [136], whereas those of *Euschistus egglestoni* can offer up to 1910 mg per 100 g [134].

### 4.3. Antinutrients 

Antinutrients are natural or synthetic compounds that reduce the bioavailability and/or utilization of nutrients when consumed in large quantities and over a long period of time. Edible insects also contain various antinutrients, which should be considered when evaluating their nutritional value and safety [137]. Common antinutrients include phytic acid [138], oxalates [139], cyanogenic glycosides [140], saponins [126], thiaminase [141], and tannins [142]. These biologically active compounds can interfere with nutrient absorption and utilization in several ways [143].

Some antinutrients, such as oxalate and phytic acid, directly chelate minerals and proteins, making them unavailable for absorption [144]. Antinutrients sometimes bind to proteins, especially digestive enzymes, which can lock nutrients in undigested food complexes [145]. Even if digestion and absorption occur, antinutrients can act as barriers to the proper utilization of nutrients in the body [146].

The presence of these antinutrients highlights the need for appropriate processing methods to mitigate their effects [147]. Thermal processing techniques such as roasting, boiling, and frying can reduce the levels of tannins and oxalates in insects [148]. Fermentation and enzymatic treatments can also decrease phytate content, enhancing mineral bioavailability [149]. For instance, the fermentation of *Macrotermes nigeriensis* is an effective means of improving its nutritional qualities by reducing antinutrients such as phytate, oxalate, and tannins while at the same time providing better conditions for degrading inhibitors of mineral absorption [150]. 

### 4.4. Digestibility and Bioavailability 

The digestibility of insect proteins appears to be high, which is essential for their potential integration into human diets. The studies highlight that insect-derived proteins, such as those from lesser mealworms, have similar digestion and amino acid absorption kinetics to milk-derived proteins, with no significant differences in postprandial muscle protein synthesis rates in humans [151]. Insect proteins are not only of high quality but also have a high digestibility (77–98%) and a favorable concentration of essential amino acids [152]. The review of edible insects as a dietary protein source for human consumption confirms their high protein content, amino acid composition, and digestibility, suggesting that they could serve as an alternative protein source [5]. In vitro studies on the digestibility of selected insect meals in poultry and quails have shown potential for certain insect species to be used as alternative protein sources, with varying digestibility coefficients for dry matter, organic matter, and crude protein [153].

The protein quality of insects has also been evaluated for potential ingredients in dog and cat food, with varying results depending on the insect species and life stages but generally indicating that insects could be a sustainable protein source for pet food [154]. A systematic review of in vivo studies on the digestibility and quality of edible insect proteins in animal models has shown mixed results, with some studies indicating similar or greater weight gain compared with control groups but also lower true digestibility and protein efficiency ratios in some cases [155]. However, a study on the digestibility of black soldier fly larvae meal in dog food found similar nutrient digestibility values to a meat-based diet, suggesting its suitability as a sustainable protein source for pet food [156].

The protein quality of insects for human consumption has been supported by limited data, indicating that farmed insect species and those harvested from the wild are likely to be of good quality, although the use of standard conversion factors may underestimate the protein quality of insects compared with other animal-source foods [157]. A study on the protein quality of mealworm larvae and crickets determined by in vitro digestible indispensable amino acid scores (DIAASs) showed that these insects have high total protein in vitro digestibility and can provide good-to-excellent protein quality, comparable to chicken breast, depending on the processing and food preparation methods used [158]. 

Furthermore, certain insects. like crickets. are noted for having comparable or even higher levels of bioavailable iron, calcium, and manganese than traditional meat sources, such as sirloin beef [159]. The bioavailability of iron from insects is particularly noteworthy; some species show higher iron solubility than beef, indicating that insects could serve as excellent sources of bioavailable iron [159]. However, Mwangi and co-workers [160] found that iron absorption from house crickets and fortified maize porridge with crickets was low, which could be explained by the presence of chitin and other inhibitors in the cricket biomass.

### 4.5. Obesity and Health Benefits 

The current global problem is obesity and over-eating, affecting over one billion people worldwide according to the World Obesity Federation [161]. Obesity-related health issues, such as cardiovascular diseases, diabetes, and certain cancers, pose significant challenges to healthcare systems globally. Addressing obesity is not just about reducing caloric intake but also improving the quality of the diet. Incorporating insects into the diet can help overcome obesity-related health problems, as they are rich in high-quality proteins and low in unhealthy fats, potentially aiding in weight management and reducing the risk of obesity-related diseases.

Insect-based proteins offer several advantages for individuals seeking to manage their weight. First, the high protein content in insects promotes satiety, helping to reduce overall food intake. Proteins are known to be more satiating than fats and carbohydrates, making them an essential component of a weight management diet. Additionally, the beneficial fats present in many edible insects, such as polyunsaturated fatty acids, contribute to heart health and can help mitigate the negative effects of unhealthy fats typically found in conventional meat sources.

Moreover, insects are low in carbohydrates and sugars, which are often linked to weight gain and metabolic disorders when consumed in excess. By replacing traditional protein sources with insects, individuals can lower their intake of saturated fats and refined sugars, promoting a healthier diet overall. The nutritional profile of insects supports muscle maintenance and growth, which is crucial for metabolic health and effective weight management. This adds another dimension to the benefits of insect-based proteins, providing a “winning formula” for addressing both nutritional needs and health challenges. The integration of insect proteins into the diet not only supports sustainable food systems but also offers a practical solution to the global obesity epidemic. By promoting insect consumption, we can enhance dietary diversity, improve public health outcomes, and contribute to a more sustainable and resilient food future.

In summary, the inclusion of insect-based proteins in the diet can play a significant role in combating obesity and related health issues. Their high protein and low unhealthy fat contents, combined with their sustainability, make insects ideal components of a health-conscious and environmentally friendly diet. This multifaceted approach addresses the dual challenges of improving public health and promoting sustainable food practices, positioning insect-based proteins as a vital element in the future of global nutrition.

## 5. Safety 

The available evidence suggests that insects are generally safe and offer beneficial or neutral outcomes compared with other foods [162]. However, some insects may produce or contain toxic bioactive compounds, and there is potential for contamination with pesticides and heavy metals from the environment [163,164,165]. Additionally, allergic reactions to edible insects could pose a hazard in some individuals [166,167]. 

Insects can harbor various pathogens, including bacteria, viruses, and parasites, which can cause foodborne illnesses (Gałęcki et al., 2023). For example, Garofalo and co-workers [168] found relatively low counts of harmful bacteria in processed edible insects, but further studies are needed to evaluate the influence of rearing and processing conditions on the microbiota. Edible insects reared under controlled conditions are expected to pose no additional hazards compared with traditional animal products [166].

Effective strategies to mitigate these risks involve implementing controlled farming practices to reduce the accumulation of heavy metals and pesticide residues [169]. Thorough processing, including washing, blanching, and cooking, can reduce microbial contamination and deactivate potential allergens. Developing and enforcing regulatory frameworks is crucial to ensuring the safe farming, processing, and distribution of edible insects [170]. 

There is a pressing need for comprehensive regulatory frameworks to ensure the safety of edible insects. These frameworks should address species selection, identifying and approving safe insect species for consumption, and farm management, establishing guidelines for safe and hygienic farming practices [171]. Regular environmental monitoring is also essential to preventing contamination. Additionally, further research is necessary to fill existing knowledge gaps. Comprehensive human studies are required to directly measure the health outcomes of insect consumption and validate the safety and health benefits of entomophagy.

Insects harbor specific viral pathogens considered safe for humans, but arthropod-borne viruses (arboviruses), like Dengue, West Nile disease, Rift Valley Fever, Hemorrhagic Fever, and Chikungunya, pose potential risks. There is also a possibility that viruses introduced into insect farms via substrates could be transferred to humans [172]. Microbial contamination remains a concern, as insects can act as vectors for pathogenic microorganisms. However, with proper processing and storage, insects can be deemed safe for consumption [41]. Insect pathogens are typically specific to invertebrates, and there is a significant phylogenetic distance between insects and humans, reducing the risk of transmission to vertebrates [173].

Research by [174] on mealworms (*Tenebrio molitor*) fed contaminated wheat bran showed that the survival of *Salmonella* sp. in larvae depends on the contamination level, suggesting competitive exclusion by endogenous microbiota and/or antibacterial activity of the larvae. Parasites are another concern, with some regions where insect consumption is traditional highlighting the transmission of trematodes and pathogenic protozoa through insects [172,175]. Mycotoxin contamination can occur when insects are improperly handled or stored. Low levels of aflatoxin B1 were found in the edible stink bug (*Encosternum delegorguei*) stored in recycled grain containers [176]. Deoxynivalenol transfer from wheat substrate to mealworm larvae was observed at high mycotoxin concentrations [177].

Feeding substrates for insects may also contain environmental contaminants, like heavy metals and chemical residues. Edible insects can accumulate heavy metals from their environment, which poses significant health risks. Lead and arsenic contamination can result from polluted soil or water where insects are harvested or farmed. Regular monitoring and stringent quality control measures are essential to ensuring that edible insects meet safety standards for heavy metal content. Notably, heavy metals such as cadmium and arsenic have been found in black soldier fly, housefly, and yellow mealworm larvae [178,179]. Veterinary drugs and pesticide residues are also concerns, with detected contaminants including nicarbazin, chlorpyrifos, and piperonyl butoxide in various insect samples [178]. 

Insects can trigger allergic reactions through contact, inhalation, and ingestion. Long-term exposure to insect antigens can cause respiratory sensitization, particularly in professional insect farmers, with insects such as grasshoppers and silkworms being common allergens [180,181]. Cross-reactions among allergens found in insects and other species, such as house dust mites, have also been documented [182,183].

Health concerns related to chitin, a component of insect exoskeletons, include potential antinutrient effects that might impact protein digestibility [167]. Despite being generally indigestible, some human gut bacteria produce chitinolytic enzymes, suggesting that chitin could be partially digested [101]. Insects also possess defensive mechanisms, including the production of irritants and toxins like alkaloids, cyanogenic glucosides, and benzoquinones, which can have significant systemic effects [172]. 

Physical dangers, particularly from insect “hairs”, spines, and the hard cuticle found in adult species, also present a concern. These physical components can cause mechanical damage to the digestive tract if not properly processed. Ensuring that insects are adequately processed to remove or soften these physical hazards is critical to consumer safety. Techniques such as grinding, milling, or removing inedible parts can help mitigate these risks.

Limited data on the toxicity of edible insects necessitate further research. Gao and co-workers [184] reviewed the toxicological characteristics of edible insects in China, noting that fewer than 34 species have been assessed, with highly variable tolerated doses having been observed in rats. This variability highlights the need for standardized toxicological evaluations. To ensure the safety of edible insects, specific HACCP procedures have been developed and applied (Ramos Fraqueza and da Silva Coutinho Patarata, 2017 [185]).

Vandeweyer and co-workers [186] assessed the microbial quality of mealworms and house crickets reared for human consumption, finding that proper processing significantly reduces microbial counts. Wynants and co-workers [174] demonstrated that blanching can effectively reduce microbial load in lesser mealworms, though some spores remain. The study by Murefu and co-workers [187] highlights the geographic distribution of research on edible insect safety, with Europe leading in publications focused on reared insects, while African research often examines wild-harvested insects. Grabowski and Klein [188], further explored the microbiology of processed insect products, noting higher microbial counts in dried and powdered insects compared with those that are deep-fried or cooked. Vandeweyer and co-workers [189] applied real-time quantitative PCR to detect antibiotic resistance genes in mealworms and crickets, revealing differences in resistance profiles between these insects. Such findings underscore the importance of ongoing research and regulation to ensure the safety of edible insects for human consumption.

## 6. Extraction and Processing of Insects

Table 2 presents a summary of the extraction and processing of insects.

### 6.1. Harvesting Methods 

The term “minilivestock” refers to insects and other small-sized organisms that can be farmed and consumed by humans [200]. While about 92% of edible insects are harvested from the wild [201], this practice raises quality and safety concerns and risks species extinction [202]. For example, the red agave worm (*Comadia redtembacheri*), the Navajo reservation ant (*Liometopum apiculatum*), and the agave weevil (*Scyphophorus acupunctatus*) are potentially endangered by overharvesting [203]. Additionally, not all insects can be successfully reared under artificial conditions [204], and pathogens can spread through captive populations, as seen with the *Acheta domesticus* densovirus (AdDNV) in commercial house cricket farming [205].

Despite these challenges, harvesting certain edible insects can be beneficial. Many insects, such as *Locusta migratoria* (Orthoptera), *Oryctes rhinoceros* (Coleoptera), and *Anaphe panda* (Lepidoptera), are agricultural pests. The manual collection of these pests can save crops and reduce the use of chemical pesticides [41].

Insect farming strategies vary. Some insects, like mealworms, cockroaches, and certain beetles, can be fully domesticated and reared in captivity, while others, like locusts, wasps, and palm weevil larvae, are semi-cultivated in modified habitats that mimic their natural environments to increase production without entirely separating them from wild populations (Feng et al., 2018). Semi-cultivation practices, such as harvesting edible eggs from artificial oviposition sites or deliberately cutting palm trees to trigger egg laying by palm weevils, contribute to habitat conservation and food security [206].

Insects like the black soldier fly (*Hermetia illucens*), common housefly (*Musca domestica*), and yellow mealworm (*Tenebrio molitor*) can efficiently convert organic waste into biomass, potentially processing 1.3 billion tons of bio-waste annually [207]. In Thailand, insect farming is significant, with 20,000 farms producing around 7500 tons of insects per year, primarily native crickets and house crickets [208]. In temperate zones, family-run enterprises rear insects such as mealworms, crickets, and grasshoppers in controlled environments to prevent desiccation [209].

Berggren, Jansson, and Low [210] compared small-scale and mass-rearing facilities. Small-scale farms, common in southeastern Asia and Africa, focus on local markets and supplement farmed insects with wild-caught individuals [211,212]. In contrast, large-scale farms, increasingly found in Western countries and Asia, rely on core breeding stocks to minimize disease introduction [41]. Transitioning from small-scale to mass production involves exploring the use of food wastes as growing substrates. Since a significant portion of global food production ends up as waste, rearing edible insects on such substrates could produce protein sustainably. Promising results have been seen with black soldier fly, house fly, mealworm, and house cricket mass production on various food wastes [213].

Collection methods for wild insects vary, as illustrated by practices in southern Cameroon, where methods include manual capture and the bucket method, depending on the insect species and their activity periods [214]. For example, nocturnal insects like crickets and termites are captured at night, while diurnal insects like honeybees are captured during the day. Overall, the safe and sustainable production of edible insects involves developing efficient rearing methods, ensuring food safety through proper processing, and integrating insect farming into circular economy models.

In the context of harvesting techniques and methods for collecting and farming edible insects, sustainable practices are essential in tropical countries where edible insects are traditionally harvested from nature. Issues such as overexploitation, habitat changes, and environmental contamination necessitate the development of sustainable harvesting practices [29]. In the central region in Cameroon, 18 species of insects are harvested by using methods such as semi-domestication, handpicking, light trapping, and net trapping, with women and teenagers being primarily involved in this activity [215].

Insect farming is emerging as a profitable enterprise in East Africa, providing a “climate-smart” protein source. This review highlights the importance of key substrates and insect species commonly farmed, the nutritional values of insects, processing techniques, and the regulatory framework. It also emphasizes the need for effective public–private partnerships to scale these technologies [52]. In the European context, edible insect farming is considered a developing business sector with the potential for significant contributions to food system sustainability. However, consumer acceptance in Western societies remains challenging, necessitating targeted marketing strategies and consumer education [55].

Lastly, edible insects in agricultural systems provide multiple ecosystem services, contributing to provision, regulation, maintenance, and cultural services. However, they can also cause significant crop damage, which is a disservice that needs to be managed. The economic and environmental contributions of these services and disservices are areas that require further research [216]. In summary, while edible insect harvesting and farming offer sustainable and nutritious alternatives to conventional meat products, several challenges must be addressed. These include developing sustainable harvesting practices, improving consumer acceptance, and managing the ecosystem services and disservices provided by insects.

### 6.2. Processing Technologies

Edible insects can be processed by using various methods, including freeze drying, sun drying, boiling, and roasting. A survey in Lagos State by Adeoye, Alebiosu, Akinyemi, and Adeniran (2014) found that roasting (62%) was the most preferred method, followed by frying (28%) and boiling (7%) [217]. To appeal to more skeptical consumers, insects can be processed into unrecognizable forms, such as ground or paste, and incorporated into products like crisps, bread, pasta, oils, beverages, and confectioneries.

Melgar-Lalanne, Hernàndez-Àlvarez, and Salinas-Castro (2019) reviewed both conventional and innovative insect processing technologies [9]. Traditional methods include sun drying, freeze drying, and oven drying, but advanced techniques such as microwave-assisted drying, bed drying, vacuum drying, and conventional hot drying on rotating racks are also used. These methods generally cause minor changes in the protein, fat, and fiber contents of insects. Various techniques have been tested for extracting proteins, fats, and chitin from insects, including water extraction, organic solvents, enzymes, dry fractionation, and sonication. Oil extraction methods include Soxhlet extraction, aqueous extraction, Folch extraction, ultrasound-assisted aqueous extraction, and supercritical CO_2_ extraction. Cold atmospheric pressure plasma can produce high-quality insect flour, although the costs are currently high [218].

Complex multi-step processes are sometimes used to prepare insects for consumption. For example, in Zambia, caterpillars are collected, eviscerated, roasted over hot coals until their adornments are burned off, sun-dried to make them crispy, and then packaged [219]. In Kenya, termites and lake flies are processed into products like crackers, muffins, sausages, and meatloaf [220]. Innovative products, such as a spicy Mexican food made from chickpeas and lesser mealworms for the Dutch market [221] and a popped snack based on mealworms and cassava in Europe [41] showcase the versatility of insect-based foods. Another notable product is the protein-enriched sorghum porridge SOR-Mite, which combines sorghum with flying termites and won a competition for developing solutions for developing countries. Recently, companies have been working on extracting and restructuring insect proteins into versatile food ingredients, such as soluble protein powders for beverages and textured proteins for meat analogues and dairy replacements [222]. Additionally, 3D printing has been proposed to enhance the aesthetics and texture of insect-based foods [223]. 

The production of edible insects is currently concentrated in household and small-scale enterprises. For the widespread adoption of edible insects as food and feed, large quantities of raw materials with standardized quality are needed, necessitating industrial-scale production. This scale-up requires the development of regulations and guidelines for producers, ensuring that products are low-cost, nutritionally valuable, and easy to store and have a long shelf life [41]. Another challenge is the high cost and availability of suitable processing equipment, which is still a relatively new area requiring substantial capital investment. Since early 2018, Bühler Insect Technology Solutions and Alfa Laval have collaborated to offer advanced modular insect plant solutions, addressing some of these challenges.

In the conversion of raw insects into consumable and marketable products, various technologies are employed to ensure safety, quality, and palatability. Initial steps often involve harvesting and pre-processing, including decontamination methods such as smoking, drying, blanching/boiling, marination, cooking, steaming, toasting, or a combination of these methods [10]. Drying is a critical process and can be achieved through methods like sun drying, freeze drying, oven drying, fluidized bed drying, and microwave drying [9]. These methods dehydrate the insects and inactivate microbes and enzymes that could spoil the product or reduce its nutritional value. After drying, further processing, such as grinding, is typically performed to produce insect meal or flour. This step is crucial to incorporating insects into food products in a non-recognizable form, which can be important for consumer acceptance in certain markets [9]. Ground insect products can then be used in various food items, like cookies, chocolates, and snacks.

Extraction processes are significant, particularly for isolating specific components such as protein, fat, or chitin. Fermentation has also been identified as a promising method to obtain insect-based ingredients with unique properties, suggesting potential for the development of innovative products [224]. Industrial processing technologies have been adapted for insect larvae de-fatting, using both dry and wet methods. These processes produce low-fat meal while recovering valuable lipids and chitin, which can be used as biomaterials [225]. The recovered fat can be an additional product, while separating chitin increases the protein content of the larvae meal.

In summary, the transformation of raw insects into marketable products involves a combination of traditional and innovative technologies aimed at ensuring microbial safety, standardizing product quality, and improving production sustainability. These technologies, ranging from drying and grinding methods to advanced extraction and fermentation processes, contribute to developing a diverse array of insect-derived food and feed products.

### 6.3. Innovation in Processing

Innovations in the processing of edible insects are essential to improving their safety, nutritional value, and consumer acceptance. Advances in technology and methods for farming, harvesting, and processing insects can significantly impact the scalability and sustainability of insect-based food production. These innovations not only enhance the quality of insect-derived products but also help address some of the challenges associated with traditional insect consumption.

One area of innovation is the domestication and semi-domestication of certain highly appreciated insect species. These efforts aim to create stable and controlled environments for breeding and rearing insects, which can lead to more consistent and high-quality outputs. For example, traditional rearing techniques for the edible Asian giant hornet (*Vespa mandarinia* Smith) have been documented by Kiewhuo et al. (2022), highlighting methods that can be adapted for semi-domestication to meet market demands while ensuring sustainability [226]. Additionally, the semi-domestication of the longhorn beetle *Thysia wallichii* has shown promise not only in terms of its nutritive value but also as a viable method for producing high-quality insect protein [227].

These domestication techniques can lead to several benefits, including improved control over breeding cycles, reduced risks of disease and contamination, and the ability to tailor the insects’ diet for optimal nutritional profiles [228]. Furthermore, domesticated insect species can be farmed by using more sustainable practices, reducing the impact on wild populations and ecosystems.

Emerging technologies, such as high-pressure processing, pulsed electric field, and cold plasma, are being explored for their potential to modify or replace conventional processing steps. These technologies are environmentally friendly and may enhance the processing efficiency, safety, and quality of insect-based products [10]. 

Enzymatic hydrolysis and high-pressure processing (HPP) are advanced methods that have been explored to enhance the functional properties of insect proteins, which are gaining attention as sustainable alternatives to traditional animal proteins. The application of these technologies aims to improve the solubility, emulsification, foaming, and gelation properties of insect proteins, which is crucial for their use in the food industry.

High-hydrostatic-pressure processing has been shown to improve the solubility of insect proteins, as demonstrated in a study on cricket and mealworm hydrolysates. The pretreatment of mealworm meal with pressurization likely induced protein denaturation and aggregation, which affected the degree of hydrolysis and functional properties [229]. Similarly, the application of HPP during enzymatic hydrolysis of soy protein isolate significantly reduced allergenicity and improved functional properties such as protein solubility and foaming activities [230].

The allergenicity of insect proteins is a concern, and studies have investigated the use of HHP coupled with enzymatic hydrolysis to reduce the immunoreactivity of mealworm proteins. The combination of HHP and enzymatic hydrolysis with Alcalase^®^ or pepsin improved the in vitro digestion of allergenic proteins, suggesting that this method could be used to produce hypoallergenic insect protein hydrolysates [231]. A review of the literature on enzymatic hydrolysis of edible insect proteins highlighted the potential to generate bioactive peptides with health-promoting properties, such as ACE inhibitory, antioxidant, and antidiabetic effects. Enzymatic hydrolysis not only enhances the nutritional value but also the techno-functional properties of insect proteins [117].

The optimization of enzymatic hydrolysis under high pressure has been shown to significantly improve the quality characteristics of *Protaetia brevitarsis seulensis* larval hydrolysates, including extraction yield, protein yield, and amino acid content [232]. The treatment period of high-pressure enzymatic hydrolysis was identified as a key factor in determining the quality of hydrolysates, with longer treatment periods generally leading to better quality characteristics [233]. The effects of high hydrostatic pressure on the technical functional properties of edible insect protein from *Protaetia brevitarsis seulensis* were also studied. High-pressure processing improved the essential amino acid index, protein solubility, and foaming capacity, indicating that high pressure can enhance the technical functional properties of insect proteins [234]. 

Lastly, the degree of hydrolysis was found to affect the techno-functional properties of lesser mealworm protein hydrolysates. While an increase in the degree of hydrolysis improved solubility and oil holding capacity, it reduced emulsifying ability, suggesting that controlled enzymatic hydrolysis is a viable method to extract high-quality protein with tailored techno-functionality [235]. 

In conclusion, advanced methods like enzymatic hydrolysis and high-pressure processing have significant impacts on the quality and safety of insect proteins, enhancing their functional properties and reducing allergenicity, which is essential for their broader acceptance and use in the food industry.

## 7. Future Directions

In the rapidly evolving field of insect farming, there are several promising directions for future research and development that could significantly enhance the industry’s contribution to food security and sustainability. One pivotal area is genetic and genomic selection, where there is a significant opportunity to apply genetic and genomic tools to improve insect strains tailored for food and feed production. This research could focus on creating strains with optimized economic and biological traits, such as increased growth rates, disease resistance, and enhanced nutritional profiles, leveraging insights from other agricultural organisms and the rapid population growth and rearing capabilities of insects [236].

Advancements in processing technologies are also critical. Developing new technologies to efficiently convert farmed insects into palatable and versatile food products is necessary. This includes innovating methods for mass harvesting, processing, and storage that maintain the nutritional quality of insect protein while ensuring food safety. Additionally, exploring the creation of new food products that incorporate insect protein in forms acceptable to consumers could significantly impact market acceptance.

Implementing circular business models could greatly benefit the insect farming industry by promoting sustainability and resource efficiency [237]. Future investigations should delve into the economic aspects of insect farming, such as cost reduction, value chain optimization, and integration into existing agricultural systems. Analyzing the economic viability and identifying strategies to foster a more circular economy within the sector are crucial [38].

Enhancing public acceptance and marketing is another essential strategy for the industry’s growth. Research should aim to understand consumer perceptions and devise effective marketing strategies to highlight the environmental and nutritional benefits of insect-based products. This may involve educational campaigns, innovative product branding, and sensory evaluations to improve consumer familiarity and acceptance.

The potential of insect farming to contribute to sustainability and circularity in the agri-food industry warrants further exploration. Research could examine the environmental impacts of insect farming, such as greenhouse gas emissions, land use, and water consumption, and compare these to traditional livestock farming. Additionally, studies could investigate the integration of insect rearing into circular agricultural practices, such as using organic waste as feed for insects [238].

Furthermore, improving insect rearing methods, both traditional and advanced, is vital to enhancing the efficiency and scalability of production. This includes optimizing rearing conditions, automating production processes, and developing new feed formulations that maximize growth and nutritional value. Exploring the role of insects in aquaculture and their potential to enhance the sustainability of fish farming is also a promising direction. By addressing these areas, the insect farming industry can advance toward a more sustainable and economically viable future, making substantial contributions to global food security and environmental conservation.

Lastly, while this study underscores the potential benefits of insect-based proteins, it is important to acknowledge its limitations, which form the basis for future research. The variability in nutritional content among different insect species and the presence of antinutrients require further investigation to optimize the health benefits of edible insects [228]. Additionally, the potential contamination with heavy metals and the physical dangers posed by insect parts necessitate more comprehensive safety evaluations and standardized processing methods [239]. Market acceptance also remains a challenge, particularly in regions where traditional insect consumption is declining or where cultural barriers exist [240]. Future research should focus on addressing these limitations by exploring the nutritional optimization of insect species, improving processing techniques to enhance safety, and developing strategies to increase consumer acceptance and market integration of insect-based foods. By addressing these gaps, future studies can build on the current findings and further support the adoption of insect-based proteins as a sustainable and nutritious component of the global food system.

## 8. Conclusions

The review highlights the substantial potential of insect-based protein to contribute to a more sustainable and secure food system. Edible insects offer numerous environmental benefits compared with traditional livestock, including lower greenhouse gas emissions, reduced land and water usage, and the ability to convert organic waste into high-quality protein efficiently. These attributes position insects as a highly sustainable protein source that can help mitigate the environmental impact of food production and support global food security. Nutritionally, insects are rich in essential macronutrients and micronutrients, making them a valuable addition to human diets. They provide high-quality protein, healthy fats, and important vitamins and minerals, which can address nutritional deficiencies and enhance overall diet quality. The digestibility and bioavailability of insect-derived nutrients further underscore their potential as a significant food source.

Safety concerns related to edible insects, such as potential contamination with pathogens, heavy metals, and allergens, can be effectively managed through controlled farming practices and thorough processing methods. Regulatory frameworks and ongoing research are essential to ensuring the safety and acceptance of insect-based foods. Processing technologies for edible insects have advanced significantly, with both traditional methods and innovative techniques being employed to produce a variety of insect-based products. These technologies enhance the safety, quality, and palatability of insect-derived foods, making them more acceptable to consumers. Case studies of global initiatives and brands demonstrate the feasibility and benefits of commercial insect farming. These examples highlight diverse applications, from animal feed to human food products, showcasing the innovative approaches taken to harness the nutritional and environmental benefits of entomophagy. Future research and development in genetic and genomic selection, processing technologies, circular business models, public acceptance, and sustainability will further enhance the viability of insect farming. By addressing these areas, the insect farming industry can advance towards a more sustainable and economically viable future, making substantial contributions to global food security and environmental conservation.

As Meyer-Rochow and Jung (2022) creatively promote, “Mealworms and spaghetti is food that makes you happy!”, and “Forget about the fork and put a cricket on your fork!” [241]. These catchy phrases capture the essence of integrating edible insects into our daily meals, highlighting their potential to bring both joy and health benefits to our dining experiences. By adopting such innovative and positive messaging, we can enhance the appeal of edible insects and encourage their acceptance on a global scale.

In summary, insect-based protein presents a promising solution to some of the most pressing challenges in food production today. Its potential to provide a sustainable, nutritious, and safe alternative to conventional protein sources is increasingly recognized, paving the way for its broader adoption in global food systems.

## Figures and Tables

**Table 1:** Summary of the nutritional contents of various edible insect species, including protein and fat percentages (dry weight), essential fatty acids, and specific amounts of vitamin B12, iron, and zinc per 100 g.

Insect Species	Protein (% Dry Weight)	Fat (% Dry Weight)	Essential Fatty Acids	Vitamin B12 (mcg/100 g)	Iron (mg/100 g)	Zinc (mg/100 g)	Ref.
Crickets (*Acheta domesticus*)	65–70	10–20	High in omega-6	5–10	5–8	3–5	[94]
Mealworms (*Tenebrio molitor*)	50–60	20–30	Balanced omega-3 and -6	0.5–1	6–9	2–3	[95]
Grasshoppers (*Locusta migratoria*)	60–75	6–8	High in omega-3	8–15	8–20	5–7	[96]
Silkworm Larvae (*Bombyx mori*)	55–65	8–12	Moderate in omega-6	2–4	10–15	3–4	[97]
Ants (*Camponotus* spp.)	45–55	14–25	High in omega-6	Trace	5–8	2–3	[98,99]

**Table 2:** Overview of various processing methodologies for edible insects, describing each method and examples of their applications.

Methodology	Description	Examples	Ref.
**Mechanical processing**			
Grinding and milling	Insects are ground into fine powders for use in various food products.	Protein bars, snacks, and pasta	[190]
Extrusion	Insect meal is pushed through a die under high pressure and temperature, creating textured products.	Textured protein products	[191]
**Thermal processing**			
Blanching	Insects are briefly boiled or steamed to kill pathogens and improve texture and flavor.	Pre-processing before further use	[192]
Roasting and baking	Methods used to enhance flavor and crunchiness of whole insects or insect-based snacks.	Snacks and whole roasted insects	[9]
**Biochemical processing**			
Fermentation	Fermenting insects to enhance their nutritional profile, digestibility, and flavor.	Fermented insect products	[193]
Enzymatic hydrolysis	Using enzymes to break down insect proteins into peptides and amino acids.	Protein supplements and functional foods	[117]
**Chemical processing**			
Defatting	Using solvents or supercritical fluids to extract fats from insects, obtaining a high-protein meal.	High-protein meal	[194]
Protein isolation	Extracting and purifying proteins from insects by using chemical solvents.	Protein isolates	[195]
**Emerging technologies**			
Ultrasound-assisted extraction	Using ultrasonic waves to enhance extraction of bioactive compounds and proteins.	Bioactive compound extraction	[196]
Microwave processing	Using microwaves to quickly and efficiently dry insects, reducing processing times and improving energy efficiency.	Dried insect products	[197]
Three-dimensional printing	Using insect-based ingredients in 3D printing to create novel food shapes and textures.	Innovative food shapes and customized textures	[198]
**Hybrid processing**			
Combination techniques	Integrating multiple processing methods to optimize quality and safety of insect-based foods.	Enhanced processing outcomes and improved food quality and safety	[199]

## Data Availability

No new data were created.
